# Plant Functions in Wetland and Aquatic Systems: Influence of Intensity and Capacity of Soil Reduction

**DOI:** 10.1100/tsw.2001.257

**Published:** 2001-11-07

**Authors:** R.D. DeLaune, S.R. Pezeshki

**Affiliations:** Louisiana State University, Wetland Biogeochemistry Institute, Baton Rouge, LA 70803, USA

**Keywords:** soil oxidation reduction, plant stress, wetlands, redox, anaerobic

## Abstract

Wetland or hydric soils, in addition to excess water and limited air-filled porosity, are characterized by anaerobic or reducing conditions. Wetland plants have developed physiological and morphological adaptations for growing under these conditions. Various methods exist for measuring plant responses to reducing conditions in wetland and aquatic environments, including assessment of radial oxygen transport, cellular enzymatic transformations, changes in root structure, and nutrient uptake. However, a gap exists in quantifying the chemical properties and reducing nature of soil environment in which plant roots are grown. The variation in reducing conditions, oxygen demand, and other associated processes that occur in wetland soils makes it difficult to truly compare the plant responses reported in the literature. This review emphasizes soil-plant interactions in wetlands, drawing attention to the importance of quantifying the intensity and capacity of reduction and/or oxygen demand in wetland soils to allow proper evaluation of wetland plant responses to such conditions.

## INTRODUCTION

Hydric or wetland soils are saturated or flooded long enough during the growing season to develop anaerobic conditions that favor the growth and regeneration of hydrophytic vegetation[1]. Plant adaptation and growth are affected by two major attributes related to the excess water in hydric soils. One is the superabundance of water for necessary physiological functions of the plant; the other is oxygen-deficiency and reducing soil conditions that seriously interfere with normal root respiration and energy production and can also result in soil microbial processes that produce substances potentially toxic to the plant.

Excess water in hydric soils affects the reactivity of inorganic redox systems that usually remain inactive in well-aerated soils. Due to the presence of excess water in hydric soils, the supply of oxygen into the soil is curtailed and facultative and obligate anaerobic microorganisms use oxidized compounds as electron acceptors for respiration, thus converting them to reduced forms. Soil reduction processes in wetlands govern plant growth and development. This review focuses on research needs associated with properly quantifying soil reduction processes for evaluating plant response in wetland environments.

## Oxidation-Reduction Potential (Redox Potential) of Wetland Soils

The easiest-to-measure change occurring in a hydric soil as a result of increased wetness is the decrease in oxidation-reduction or redox potential[2]. Aerated soils have characteristic redox potentials in the range of +400 to +700 mV; flooded or waterlogged soils exhibit potentials as low as -250 to -300 mV. In wetland soils, several factors combine to make the oxidation-reduction potential the best available measure of the oxidation or reduction status of the soil. First, the range of potential in anaerobic soils is much wider, approximately 700 mV as compared to a range of approximately 300 mV in well-drained soils (Fig. 1). Second, oxygen is usually absent from most waterlogged soils; therefore, methods used for the measurement of oxygen content and oxygen diffusion rate employed in well-drained soils cannot be used in waterlogged soils.

The various inorganic redox systems found in soil become unstable at critical redox potential (Fig. 1). Sequentially, oxygen is reduced first, followed by nitrate and oxidized manganese compounds, and then ferric iron compounds. Following the reduction of ferric iron, the next redox element to become unstable is sulfate, followed by the reduction of carbon dioxide to methane.

Soil redox potential represents an indication of the oxidation-reduction status of various redox couples. For example, a redox potential of 0 mV indicates that oxygen and nitrate are not likely to be present and that the bioreducible iron and manganese compounds are in a reduced state. At this same potential, however, sulfate is stable in the soil with no production of sulfide, which is toxic to plants. A redox potential of +400 mV indicates that oxygen may be present even though there may be excess water.

## Intensity and Capacity of Reduction

Reduction of the inorganic redox system in wetland soils can be described in terms of intensity and capacity[3]. Reduction intensity factor determines the relative ease of the reduction and is represented by the free energy of the reduction, or equivalent electromotive force of the reactions. Soil redox potential, or Eh, is used to quantify the intensity of reduction. Capacity of soil reduction describes the quantity of redox species undergoing reduction and is equivalent to the total amount of electrons accepted by the soil oxidants in microbial respiratory activity. Capacity is also related to the total amount of labile carbon (C) compounds or total energy sources that are utilized during microbial activity (reductant capacity) and is described in terms of its $O_2$ equivalent. The capacity factor of soil reduction reflects soil $O_2$ demand, in addition to the soil's phytotoxin capacity and production rate[4]. Soils with the same reduction intensity may differ with respect to their capacity. Increased reduction capacity at the same reduction intensity generally leads to significant changes in plant responses. In aquatic or wetland soil systems where there is biological activity and where several redox systems function, redox potential is used to denote intensity of reduction.

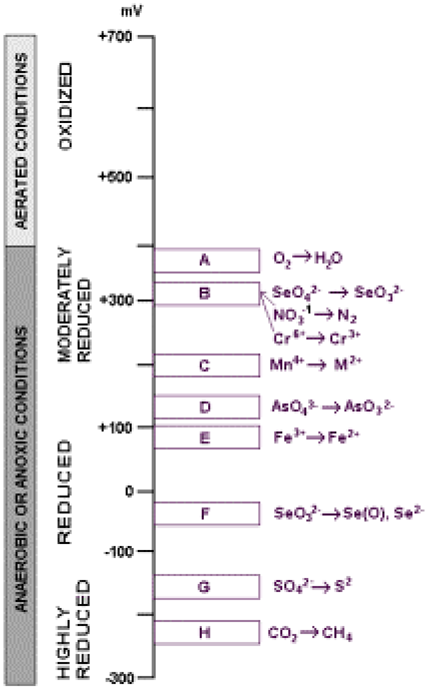

**FIGURE 1.** Critical redox potentials (at pH 7) for transformations of inorganic species. Data for A from Reddy and Patrick[5] and Turner and Patrick[6]; B from Patrick[7] and Buresh and Patrick[8]; C from Gotoh and Patrick[9]; D from Masscheleyn et al.[10,11]; E from Gotoh and Patrick[12]; F from Masscheleyn et al.[13]; G from Connell and Patrick[14], and H from Masscheleyn et al.[15].

Capacity of the various redox systems can vary from one soil to another. The amount of oxygen in the soil at the time of flooding of a well-drained soil is very low, consisting of the oxygen in the trapped air spaces plus that dissolved in the water occupying the pore spaces. The quantity of nitrate present at flooding typically is more variable than oxygen, but is usually only a few parts per million. Reducible manganese oxides are present in much higher concentrations in most soils than oxygen or nitrate, but the concentrations are variable, with some soils having less than 100 ppm reducible manganese and others having over 10 times as much. Most soils have much higher amounts of reducible iron compounds than of any other inorganic redox component. Sulfate is a variable component, with coastal salt marshes having a high concentration of sulfate and some nonsaline interior wetlands being very low in sulfate. The redox systems (Fig. 1) can be ranked on the basis of ease of reduction from the oxygen-water system to the carbon dioxide-methane system. Oxygen readily accepts electrons from decomposing plant material, whereas the reduction of carbon dioxide to methane occurs only under very reduced anaerobic conditions.

## Interpretation of Plant Response to Wetland Soil Condition

Studies dealing with responses of hydrophytic vegetation to reduced soil oxygen have utilized experiments in which plants were grown hydroponically and pressurized nitrogen was passed through the solution to remove oxygen. Roots in such systems were exposed to redox potential only slightly below values where oxygen disappears on the redox scale (i.e., +350 to +400 mV)[3]. Anaerobic conditions are values between +400 and -300 mV. Redox potential of -300 mV may occur in highly reduced soils. Since oxygen is absent at redox potential values at or below +350 mV, the absence of oxygen alone does not provide much information on the intensity of reduction. Even studies designed to evaluate responses of plants grown at the upper portion of the anaerobic range of the redox scale may yield results that are not typically the same as those exhibited by plants grown in a more reducing natural environment.

To evaluate the response of hydrophytic vegetation to oxygen-deficient soils, methods are needed to properly quantify and document the substrate condition in which the plants are grown. The common laboratory conditions that plants are subjected to may be sufficient for evaluation of the physiological response of flood-sensitive plants to low oxygen. Most laboratory methods commonly used for removing oxygen from root zones reduce redox potential to levels at which flood-sensitive species should respond to oxygen deficiency. However, such levels are not effective for studies of flood-tolerant wetland species, many of which can withstand extreme reducing conditions covering a significant portion along the redox scale normally found in wetland and coastal soils.

## Plant Responses to Soil Waterlogging

Plants use various strategies to cope with soil flooding, including morphological/anatomical responses such as adventitious roots, lenticel formation, development of an aerenchyma system allowing oxygen diffusion from aerial parts to the roots, changes in root metabolism aimed at producing the energy for survival, and acceleration in anaerobic fermentation[16]. Review of the literature concerning this relatively broad area can be found in articles by Hook and Crawford[17], Kozlowski[18,19,20,21], Drew[22,23,24], Armstrong and Armstrong[25,26], Perata and Alpi[27], Pezeshki[28], and Vartapetian and Jackson[29]. However, a few of the points pertinent to the present review will be discussed herein.

## Root Functions

Much of the immediate flood-injury to roots is attributed to anaerobic conditions[22,23,30]. Among mechanisms developed to cope with such conditions are root morphological/anatomical responses that facilitate root oxygenation and have been attributed to flood-tolerance in many species[18,21,31,32,33,34]. Adventitious roots and lenticel formation are important characteristics in flood-tolerance of many species, including herbaceous and woody species[31,32,33,35,36,37]. Stem and root lenticels are among the means by which oxygen is supplied to the flooded roots[32,35,38]. Development of adventitious roots and stem lenticels has been reported for woody species when subjected to flooding[33]. Aerenchyma tissue development is important because it facilitates diffusion of oxygen to the roots, allowing some aerobic respiration[39,40,41] and helping to detoxify a reduced rhizosphere[39,42]. Aerenchyma formation in some species appears to result from development of hypoxic conditions in the roots followed by enhanced synthesis and accumulation of ethylene[43,44]. It allows passage of air via diffusion between above- and belowground portions of the plant. Although diffusion is a major pathway of root aeration in wetland plants, it is not the only one. Ventilation in rhizomes due to pressurized throughflow of gases also has been reported for some species, as has the venturi-induced convection pathway[45,46,47,48,49].

Clearly, normal growth and functioning of roots requires more oxygen than is needed simply for root respiration[28,30,50]. Under aerated conditions, oxygen diffuses into the roots from soil air spaces via the root epidermis. However, when roots are under flooded conditions, the required oxygen must reach the roots through internal paths from the aerial parts[51]. Most wetland plants develop an extensive aerenchyma system extending from substomatal cavities to the roots[52,53,54]. In many emergent species, too, oxygen enters the plant through stomatal pores as well as through lenticels[55]. In a typical wetland plant, the presence of aerenchyma tissue has been reported in various plant organs including stems, petioles, leaves, and rhizomes[56].

The development of morphological and anatomical root adaptations in response to oxygen stress is time dependent; that is, it may take up to several weeks before these systems are fully developed and functional. Thus, during the initial period of stress, the required energy for survival is generated through anaerobic metabolism[30,57]. The general consensus is that plants tolerate anaerobic conditions by accelerated ethanol fermentation in the roots. It has been shown that many species rely on anaerobic metabolism as a means of surviving anaerobic root conditions[58,59]. The enzyme involved in catalyzing the reaction that produces ethanol, alcohol dehydrogenase (ADH), is found at a high concentration in roots of flood-tolerant plants under flooded conditions[30,40,58,60]. The role of ADH in flood-tolerance has been known for some time[29], although its specific functions are still being debated. These functions include maintaining intracellular pH and cellular energy requirements[55,61].

Elevated tissue ethylene concentration has been found under flooded conditions[62,63]. The effect of increased ethylene concentrations on roots includes enhancing aerenchyma formation in certain species[43,64,65]. Ethylene can also inhibit root elongation[66,67] and, sometimes, inhibit the elongation of stems[68]. This promotion results from enhanced cell growth, increased cell numbers, and increased cell wall acidification[69,70]. The involvement of ethylene and abscisic acid in the flood-response of plants has been documented; however, the possible role of other growth regulators needs further investigations[63]. For example, the balance between auxins transported from the shoot and root-produced substances may be critical under anaerobic soil conditions, as was pointed out by Schumacher and Smucker[71]. It is generally believed that most known plant hormones can influence root growth to different degrees[72].

Most of the root-response studies mentioned above lack information on substrate redox conditions (intensity and capacity of reduction), oxygen demand, and presence and/or concentrations of reduced compounds (e.g., sulfide) that plant roots were likely exposed to during the studies. It is clear that such information would be useful to plant scientists for predicting competitive ability of wetland plant species and adaptation limits for growing in aquatic environments.

### Root Oxygenation

Root oxygenation is an important adaptation that helps plants overcome intense anaerobiosis[22,23,25,73,74] and has important ecological implications in wetlands. For example, in coastal wetlands the distribution of *Spartina alterniflora* into more regularly flooded marsh habitats than *S. patens* is due to the more efficient $O_2$ transport to the roots of *S. alterniflora*[75]. In addition, the vigor and productivity of *S. alterniflora* was found to be positively correlated with substrate redox potentials because of the interaction with root aeration[76,77,78,79]. Two interrelated factors probably limit growth under highly reduced conditions: (1) the lower redox levels represent an $O_2$ deficient system and (2) phytotoxins accumulate to a level where roots oxidizing power no longer can ameliorate their effects[78,80,81,82]. The roots must rely more heavily on anaerobic respiration[35,83] or transport sufficient oxygen to roots to maintain aerobic respiration, lessening its capacity to oxidize the rhizosphere[84,85]. Root ADH increase in *S. alterniflora* with decrease in sediment redox potential has been reported[86].

Aerenchyma, which provides a major pathway for transporting oxygen to the roots, represents an energy-efficient adaptation that avoids the problems of root anaerobiosis[16]. It allows less resistance to $O_2$ movement for respiring cells[39], and decreases the amount of respiring tissue while still providing structural support[42]. The variation in flood-tolerance has been associated with the resistance to air movement across the vascular cambium[87], survival of secondary roots, development of new secondary roots and adventitious roots, accelerated anaerobic respiration, and rhizosphere oxidation[21,29,36,56].

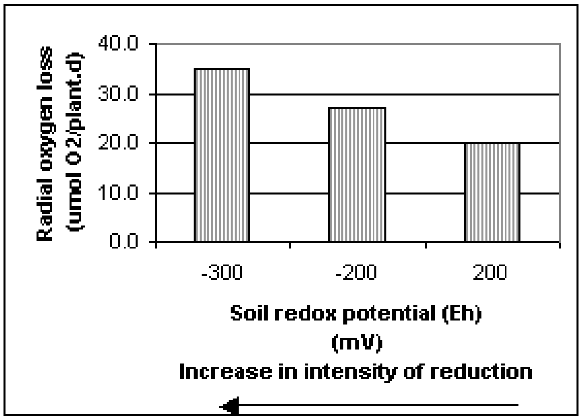

**FIGURE 2A.** Radial $O_2$ loss from rice as a function of soil redox potential or intensity of reduction. (Modified and redrawn from Kludze et al.[93].)

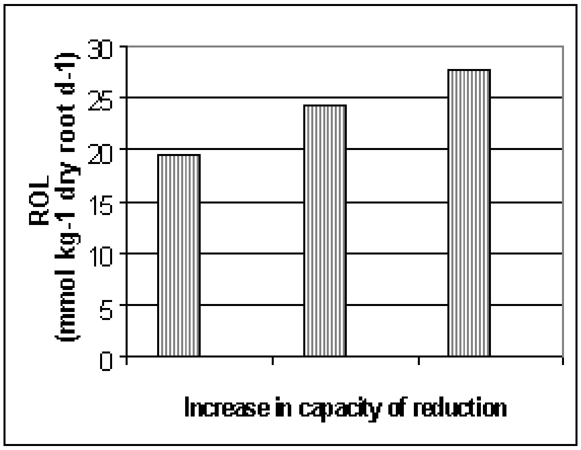

**FIGURE 2B.** Radial oxygen loss from rice in response to change in soil reduction capacity. (Redox intensity was maintained at -200 mV.) Capacity was increased by glucose additions, A = 0 g $kg^{-1}$, B = 0.08 g $kg^{-1}$, C = 0.16 g $kg^{-1}$ glucose. (Modified and redrawn from Kludze and DeLaune[4].) The different letters represent significant differences at the 0.05 level using Tukey's test.

## Rhizosphere Oxygen Demand

Most wetland plants are well-adapted to periods of soil oxygen deficiency but may differ in their ability to endure intense soil-reducing conditions[88,89,90,91]. Such conditions create the potential for excessive loss of oxygen from the root to the soil, thus resulting in additional root stress[4,88,92]. DeLaune et al.[88] used titanium citrates as a reducing agent to demonstrate that oxygen-depleted nutrient solution commonly used to evaluate plant response to root oxygen stresses are a poor analogue of wetland soil and sediment. The oxygen-depleted nutrient solution does not create a high root oxygen demand. A study by Kludze et al.[93] was the first to document that a solution of high oxygen demand (using titanium citrate) also influenced oxygen transport and oxygen release by the root system of wetland plants. Other researchers have since made similar observations[93,94]. Radial loss of oxygen by rice roots was strongly influenced by intensity of reduction in anaerobic soil (Fig. 2A). Radial oxygen loss has also been shown to be governed by intensity of soil reduction (Fig. 2B). Radial oxygen loss from rice was shown to increase by increasing capacity of reduction by glucose addition when redox intensity was maintained at -200 mV[4]. Such research has demonstrated the importance of creating an oxygen demand in the root rhizosphere for quantifying or evaluating plant physiological functions, including root oxygen exchange. However, it should be noted that the use of titanium citrate as a reducing agent for creating a reducing medium at best only mimics wet-soil conditions. Methods are needed for evaluating plant responses (including oxygen exchange in the root rhizosphere) to a quantifiable reducing soil condition since plants growing in soil are influenced by both intensity and capacity of reduction.

Studies aimed at quantifying responses of wetland plants to flooded soil conditions should distinguish between plant responses to the absence of oxygen and the responses to intensity and/or capacity of soil reduction. Kludze and DeLaune[4] conducted an experiment under laboratory conditions, demonstrating that increasing the capacity of soil reduction at any intensity level subjected wetland plants to increased stress. *Oryza sativa* (rice) and *S. patens* were grown under controlled Eh levels of 100, 0, -100, and -200 mV to examine the effect of Eh on plant $CO_2$ fixation. Treatments were established by application of different levels of extra energy source while maintaining Eh at -200 mV. Redox capacity effects on plant growth, $CO_2$ fixation, root porosity (POR), and radial oxygen loss (ROL) were also evaluated. In both species, $CO_2$ fixation did not respond to soil Eh until Eh reached values around -100 mV or lower (Fig. 3A). Although POR was unaffected, plant growth and $CO_2$ fixation were significantly decreased, with increased soil $O_2$ demand, suggesting a complex relationship between soil redox capacity and plant physiological functions (Fig. 3B). Plant $O_2$ transport to the root environment (ROL) also was governed by soil redox capacity. Results indicated that wetland plants may respond differently in magnitude to soil redox intensity and redox capacity. Evaluating responses, especially ROL, of flood-tolerant plants, therefore, requires proper quantification of the soil redox condition or substrate $O_2$ demand in which the plants are grown. Such interactions are important in controlling species diversity and distribution in wetlands; an understanding of these relationships is also important to wetland maintenance and restoration.

## Nutrient Uptake

Nutrient uptake by wetland or rooted aquatic vegetation is also influenced by soil reduction or redox conditions or intensity of reduction. A laboratory study of $^{15}N$ uptake by cherrybark oak (*Quercus falcata* var. *pagodaefolia* Ell.) and overcup oak (*Q. lyrata* Walt.) in soil suspensions under controlled redox conditions indicated that soil redox conditions governed both plant photosynthetic rates and N uptake[95]. Nitrogen uptake indicated that although available nitrogen was present in the soil solution, there was little uptake of either fertilizer N or native soil N under moderately reducing conditions (+340 and +175 mV). These results demonstrated that soil reduction intensity affected growth of both species through reduced uptake of nitrogen. Flooding of forests for extended periods of time during the growing season can disrupt the physiological functioning and nutrient uptake.

DeLaune et al.[96] documented that redox conditions or oxygen demand in rooting medium influenced phosphorus (P) uptake by *Typa domingensis*. Phosphorus uptake decreased with decrease in redox potential or reduction intensity in the rooting medium. Greatest uptake was measured under the oxidized treatment (+565 mV). Phosphorus uptake was less under two reducing treatments, and considerably less at -200 mV, in which a high oxygen demand was created using titanium ($Ti^{3+}$) citrate. Results suggest that nutrient uptake by wetland plants is governed by soil reduction intensity and capacity. This suggests that measured physiological responses in wetland plants may not be entirely or directly associated with flooding effects on plant function, but may also be associated with secondary effects such as changes in nutrient uptake.

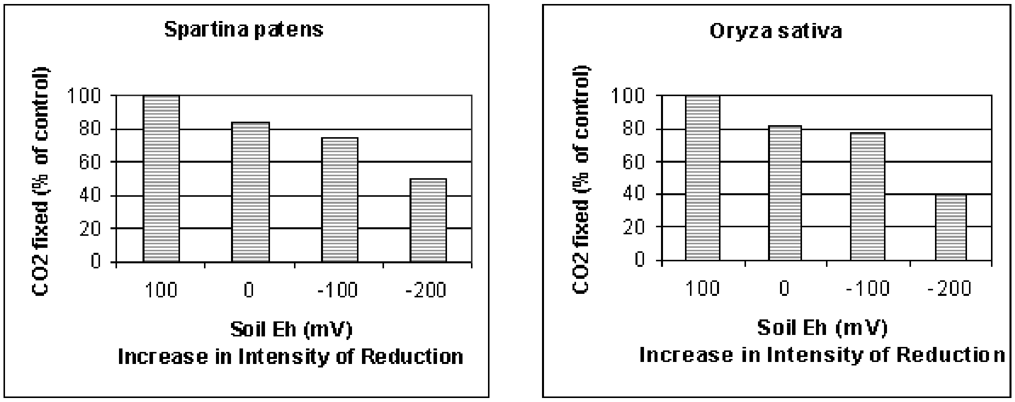

**FIGURE 3A**. Carbon dioxide fixation in saltmeadow cordgrass and rice, as a function of soil redox intensity (Eh) along the anaerobic portion of the redox scale. (Modified and redrawn from Kludze and DeLaune[4].)

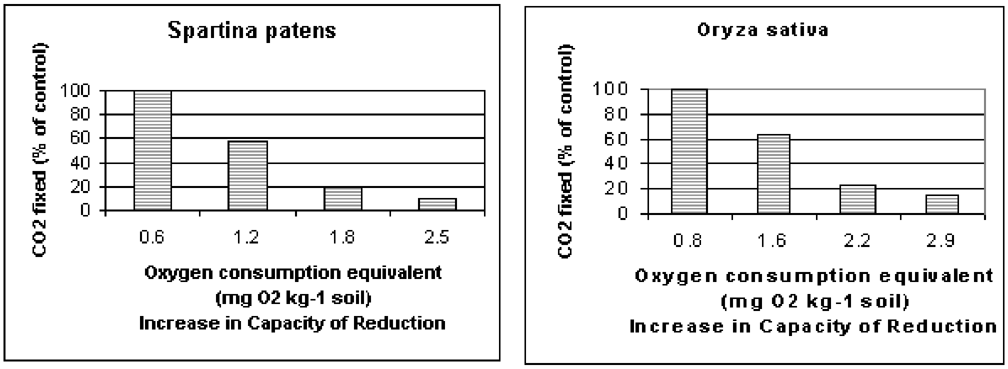

**FIGURE 3B**. Decreases in $CO_2$ fixation in saltmeadow cordgrass and rice, as a result of increasing soil redox capacity by adding extra energy source, while maintaining the soil redox intensity at -200 mV. (Modified and redrawn from Kludze and DeLaune[4].)

## Leaf Functions

Flooding and the accompanying root hypoxia may lead to leaf area reduction[97,98,99] and foliage injury, and may threaten survival and growth of plants[18,19,20,21]. Among the early responses of plants to soil oxygen depletion are plant gas-exchange responses. Most species display rapid stomatal closure and reduction of net photosynthesis in response to soil flooding[28,100,101,102]. This is a common response among species found in various flood-tolerance categories, ranging from “least tolerant” to “most tolerant.” However, net photosynthesis in wetland (most tolerant) species begins to recover rapidly following the initial reduction, whereas little or no recovery is found in least tolerant species[103]. This response is attributed to the existing tolerance mechanisms, such as rapid aerenchyma development, lenticel formation, metabolic adaptation, and other attributes found in tolerant species. In addition, there is a wide range of inter- and intra-species difference in photosynthetic responses of plants to flooding. The mechanisms involved are poorly understood. The explanation involving stomatal (diffusional) limitations and metabolic effects may account for the differences. The metabolic processes affected may include reduction in activity of photosynthetic enzymes. The activity of these enzymes is highly sensitive to changes in environmental conditions[103,104,105,106].

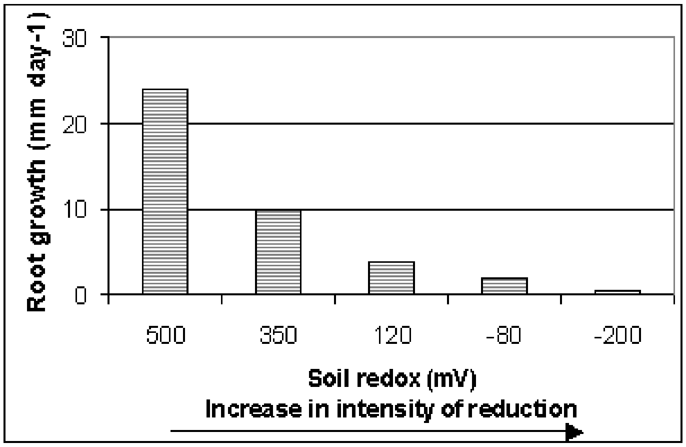

**FIGURE 4.** Root growth response to soil redox conditions in *S. patens*. Data were collected on plants grown in controlled soil redox conditions in rhizotrons. (Redrawn from Pezeshki and DeLaune[108].)

## Plant Growth and Productivity

Biomass accumulation rate decreases in response to low soil redox potential in many wetland species[4,28,89]. Significant alterations have also been reported in root-to-shoot ratios, as the effects of soil reduction are usually more dramatic on roots than shoots[85,107]. For example, root and shoot dry weights in *S. patens* decreased by 40 and 25% as soil redox potential dropped from +200 to -300 mV, respectively. It was also demonstrated that roots were more sensitive to redox intensity than shoots[4]. Pezeshki and DeLaune[108] reported significant reductions of root growth in *S. patens* at soil Eh of -100 mV (Fig. 4). In addition, Pezeshki et al.[109] noted smaller root systems in *S. patens* under reducing conditions and concluded that such reduction in sink size may, in part, be responsible for a negative feedback inhibition of photosynthesis, thus causing further reductions in productivity of this species.

Root growth is an energy-dependent process requiring oxygen; therefore, upon flooding, root functioning is affected rapidly because molecular oxygen is required as an electron acceptor for oxidative phosphorylation[22,110]. Root elongation was also inhibited in some woody species when soil redox potential measurements confirmed the presence of reducing conditions[85,89]. Root penetration depth was also adversely affected under reducing soil conditions, leading to the development of a shallow root system different in architecture than in plants growing under aerated conditions[85]. The critical threshold redox potential that inhibited root elongation differed among wetland species ranging from +300 to -200 mV[85,111,112].

Soil redox capacity also influences growth of wetland plants. Decreased soil redox capacity led to decreased root growth and biomass in rice[4]. Root and shoot growth were significantly inhibited in *S. patens* under increasing soil reduction capacity. Root and shoot dry weights decreased by 70 and 37% in high reduction capacity conditions compared to control plants, respectively[4].

## Relation to Natural Distribution of Aquatic or Wetland Plant Species

Wetland plant species are commonly found along environmental gradients[113,114]. The zonation of plant species is based, to a degree, on flooding regimes. Considerable research in recent years has been directed at determining the environmental factors delineating the boundaries or species zones. One of the most conspicuous factors along these gradients is water depth. Flood-tolerance has been shown to be a dominant factor in determining the zonation of wetland plants[51]. However, very few studies have addressed the intensity and capacity of soil reduction in relation to the zonation of wetland species along flooded gradients. It is not clear whether wetland plants compete with each other based on their differences in physiological adaptation to intensity or capacity of reduction, especially in soils that are constantly waterlogged.

Frequency of occurrence and diversity of wetland plant species may also be dependent on the interrelationships among intensity and capacity of soil reduction and root aeration capacity rather than flooding regime alone. Armstrong et al.[115] reported field data on the relationship between soil redox potential and plant community distribution in saltmarshes. In sediments characterized by weak redox capacity, certain wetland plants are capable of raising considerably the redox potential of the bulk sediment[116,117,118]. Furthermore, accumulation of various soil phytotoxins, which are by-products of soil reduction, may lead to injury to certain species. Since wetland plants are classified by frequency of occurrence in wetlands, their distribution is likely strongly influenced by both intensity and capacity of soil reduction, and ability of wetland plant species to maintain an oxygenated root environment and to take up nutrients. This is supported by the observation that wetland vegetation can differ over a range in taxonomic soil series that exhibit similar flooding regimes or water table fluctuations but differ in soil biological oxygen demand, as reflected in soil organic carbon content. It is clear that much remains to be learned about the underlying soil processes and the mechanisms of plant responses in wetlands. Specifically, elucidating the interrelationships between soil reduction intensity and capacity and soil phytotoxins in the rhizosphere and root internal processes and functioning deserve immediate attention.
